# Long read alignment based on maximal exact match seeds

**DOI:** 10.1093/bioinformatics/bts414

**Published:** 2012-09-03

**Authors:** Yongchao Liu, Bertil Schmidt

**Affiliations:** Institut für Informatik, Johannes Gutenberg Universität Mainz, Mainz 55099, Germany.

## Abstract

**Motivation:** The explosive growth of next-generation sequencing datasets poses a challenge to the mapping of reads to reference genomes in terms of alignment quality and execution speed. With the continuing progress of high-throughput sequencing technologies, read length is constantly increasing and many existing aligners are becoming inefficient as generated reads grow larger.

**Results:** We present CUSHAW2, a parallelized, accurate, and memory-efficient long read aligner. Our aligner is based on the seed-and-extend approach and uses maximal exact matches as seeds to find gapped alignments. We have evaluated and compared CUSHAW2 to the three other long read aligners BWA-SW, Bowtie2 and GASSST, by aligning simulated and real datasets to the human genome. The performance evaluation shows that CUSHAW2 is consistently among the highest-ranked aligners in terms of alignment quality for both single-end and paired-end alignment, while demonstrating highly competitive speed. Furthermore, our aligner shows good parallel scalability with respect to the number of CPU threads.

**Availability:** CUSHAW2, written in C++, and all simulated datasets are available at http://cushaw2.sourceforge.net

**Contact:** liuy@uni-mainz.de; bertil.schmidt@uni-mainz.de

**Supplementary information:** Supplementary data are available at *Bioinformatics* online.

## 1 INTRODUCTION

Many biological applications of next-generation sequencing (NGS) require the alignment of large quantities of produced reads to a given reference genome. Consequently, a wide variety of short read aligners have been developed in recent years. They can be classified into two categories according to their approaches to identify seeds: hash tables and prefix/suffix tries. MAQ (Li *et al*., 2008), SOAP (Li *et al*., 2008), SHRiMP (Rumble *et al*., 2009) and BFAST (Homer *et al*., 2009) are examples of the hash table approach. Bowtie (Langmead *et al*., 2009), BWA (Li and Durbin, 2009), SOAP2 (Li *et al*., 2009) and CUSHAW (Liu *et al*., 2012) implement the concept of prefix/suffix tries using the Burrows–Wheeler transform (BWT) (Burrows and Wheeler, 1994) and the FM-index (Ferragina and Manzini, 2005).

With the progress of NGS technologies, the length of produced reads continues to increase. Unfortunately, many existing short read aligners are becoming inefficient as generated reads grow to a few hundred bp in length because of two reasons. First, they typically perform only ungapped alignments or gapped alignments allowing a very limited number of gaps (typically one gap). Second, their speed degrades rapidly as the number of gaps increases. However, for long read alignment, more gaps must be allowed as indels will occur more frequently. These new features of long read alignment thus motivate the design of new aligners with fast speed and high quality.

In this article, we devise a new long read aligner based on the well-known seed-and-extend heuristic. This heuristic is based on the observation that significant alignments are likely to include homologous regions, containing exact or inexact short matches between the two sequences. It generally works in three steps. First, seeds, represented as short matches indicating highly similar regions, are generated between the query and the target sequences. Secondly, these seeds are extended and refined under certain constraints, such as minimal percentage identity and extension length, to filter out noisy seeds. Finally, more sophisticated algorithms, such as the Needleman–Wunsch algorithm (Needleman and Wunsch, 1970) or the Smith–Waterman (SW) algorithm (Smith and Waterman, 1991), are employed to obtain the final alignments. Several types of seeds have been proposed, including fixed-length seeds, maximal exact matches (MEMs), maximal unique matches (MUMs), and adaptive seeds (Kielbasa *et al*., 2011). Fixed-length seeds (*k*-mers) are the most widely used seed type. The simplest fixed-length seed is the exact *k*-mer match. Some improvements have been suggested by allowing mismatches and gaps in the *k*-mers, including spaced seeds (Ma *et al*., 2002), and *q*-gram (a substring of *q* bases) filters (Rasmussen *et al*., 2006). MEMs are exact matches that cannot be extended in either direction without allowing a mismatch. MUMs are inherently MEMs but require uniqueness in addition. An adaptive seed has a variable seed length, and also has a limitation on the number of seed occurrences in the target.

Recently, several long read aligners have been developed based on the seed-and-extend approach, including BWA-SW (Li and Durbin, 2010), Bowtie2 (Langmead and Salzberg, 2012), and GASSST (Rizk and Lavenier, 2010). BWA-SW, inspired by BWT-SW (Lam *et al*., 2008), identifies long gapped seeds by employing a prefix directed acyclic word graph (implicitly represented by an FM-index) to perform dynamic programming (DP). Subsequently, it heuristically extends and refines the long gapped seeds to produce the final alignments. Bowtie2 extracts all mismatch-allowable fixed-length seeds from a read using the BWT and then employs DP to identity alignments. GASSST employs hash tables to find fixed-length seeds and employs multiple filters to remove noisy seeds, prior to the final DP-based alignment. This approach is effective to significantly reduce the number of noisy seeds, but also has the risk of discarding correct ones.

In this article, we present a new long read aligner using MEMs as seeds. MEMs have been used for whole genome alignment (Bray *et al*., 2003; Choi *et al*., 2005; Delcher *et al*., 1999; Höhl *et al*., 2002). However, to the best of our knowledge, MEMs have not been used for NGS read alignment. Our aligner employs memory-efficient versions of the BWT and FM-index data structures to generate MEM seeds for each read. Each seed defines a potential mapping read region on the genome. We then compute the optimal local alignment score between the read and each potential mapping region and select the highest-scoring mapping region to produce the final alignment. In addition, our aligner provides support for paired-end (PE) alignment. For the PE alignment, a new seed-pairing approach is introduced with the intention to quickly determine the potential mapping regions of a PE read pair without performing alignments. Furthermore, we employ vectorization and multithreading to achieve fast execution speed on standard multi-core CPUs. The performance of our aligner is assessed and compared with BWA-SW, Bowtie2 and GASSST, by aligning simulated and real datasets to the human genome. The experimental results show that our aligner achieves favorable alignment quality, highly competitive speed and good parallel scalability with respect to the number of threads. This new aligner has been integrated into our software package CUSHAW. The first version of CUSHAW was designed for short read alignment (≤128-bp reads) using GPU computing. It uses mismatch-allowable fixed-length seeds and does not provide support for gapped alignments. We name the aligner presented in this article, CUSHAW2, to indicate the extended functionality.

## 2 METHODS

**Fig. 1.**
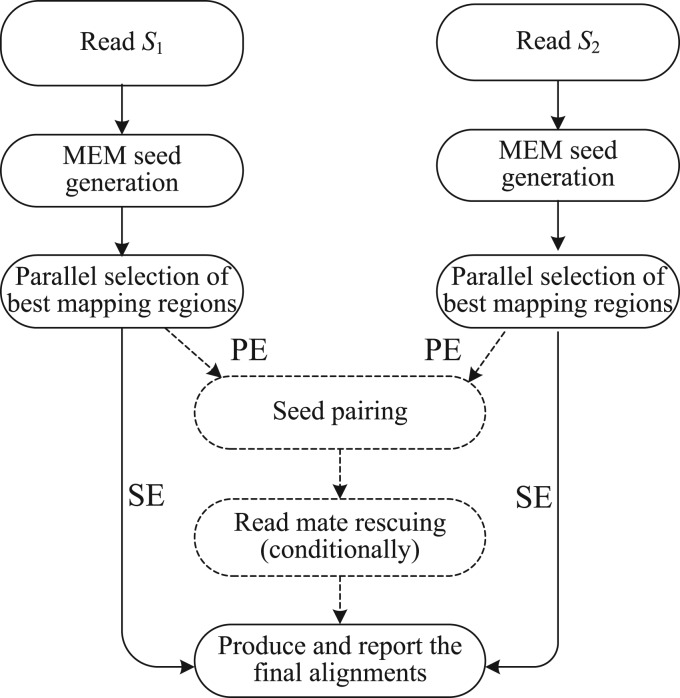
Pipeline of our aligner for the SE and the PE alignment: the dashed lines show the additional two stages for the PE alignment

CUSHAW2 employs MEMs as seeds to compute a gapped alignment for a long read to a given reference genome. For the single-end (SE) alignment, CUSHAW2 works in three stages: (i) generate MEM seeds, (ii) select the best mapping regions on the genome and (iii) produce and report the final alignments. For the PE alignment, we introduce two additional stages before producing the final alignments: one is the seed pairing stage and the other is the read rescuing stage. Figure 1 illustrates the pipelines of our aligner for both the SE and the PE alignment.

### 2.1 Essentials for maximal exact match identification

#### 2.1.1 Definitions and notations

Given a sequence *S*, define |*S*| to denote the length of *S*, *S*[*i*] to denote the character at position *i* and *S*[*i*,*j*] to denote the substring of *S* starting at position *i* and ending at position *j*, for 0≤*i* < |*S*| and 0≤*j* < |*S*|. We represent an exact match between two sequences *S*_1_ and *S*_2_ as a triplet (*p*, *q*, *k*), where *k* is the length of the exact match and the substring *S*_1_[*p*, *p* + *k*− 1] is identical to the substring *S*_2_[*q*, *q* + *k*− 1]. An exact match is called right maximal if *p*+ 1 = |*S*_1_| or *q*+ 1 = |*S*_2_| or *S*_1_[*p* + *k*] ≠ = *S*_2_[*q* + *k*], and left maximal if *p*= 0 or *q*= 0 or *S*_1_[*p*− 1] ≠= *S*_2_[*q*− 1]. An exact match is called a MEM if it is both left maximal and right maximal.

Given a sequence *T*, defined over the alphabet Σ = {A, C, G, T}, the suffix array *SA* of *T* stores the starting positions of all suffixes of *T* in lexicographical order. In other words, *SA*[*i*] = *j* means that the *i*th lexicographically smallest suffix (among all suffixes of *T*) starts at position *j* in *T*. The *SA* of *T* has an overall memory footprint of |*T*|⌈log_2_|*T*|⌉ bits (~12 GB for the human genome). Given a substring *S* of *T*, we can find all its occurrences within an *SA* interval. An *SA* interval is an index range [*I_a_*, *I_b_*], where *I_a_* and *I_b_* represent the indices in *SA* of the lexicographically smallest and largest suffixes of *T* with *S* as a prefix.

#### 2.1.2 BWT and FM-index

The BWT of *T* starts from the construction of a conceptual matrix *M_T_*, whose rows are all cyclic rotations of a new sequence *T*$ sorted in lexicographical order. *T*$ is formed by appending the special character $ to the end of *T* that is lexicographically smaller than any character in Σ. After getting *M_T_*, the last column of the matrix is taken to form the transformed text *B_T_*, i.e. the BWT of *T*. *B_T_* is a permutation of *T* and thus occupies the same memory size of |*T*|⌈log_2_ |Σ|⌉ bits as *T*. *M_T_* has a property called ‘*last-to-first column mapping*’, which means that the *i*th occurrence of a character in the last column corresponds to the *i*th occurrence of the same character in the first column.

The FM-index consists of a vector *C*(•) and an occurrence array *Occ*(•), constructed from *B_T_*, to accomplish substring search. *C*(•) contains |Σ| elements with each element *C*(*a*) representing the number of characters in *T* that are lexicographically smaller than *a* ∈ Σ. *Occ*(•) is an array of size |*T* |×|Σ| with each element *Occ*(*a*, *i*) representing the number of occurrences of *a* ∈ Σ in *B_T_* [0,*i*]. In terms of memory overhead, *C*(•) requires only |Σ|⌈log_2_ |Σ|⌉ bits but *Occ*(•) requires |Σ| |*T*|⌈log_2_ |*T*|⌉ bits.

Given a substring *S* of *T*, the *SA* interval of all its occurrences can be computed in *O*(|*S*|) time using a backward search procedure. Based on *C*(•) and *Occ*(•), the *SA* interval [*I_a_*, *I_b_*] can be recursively calculated, from the rightmost to the leftmost suffixes of *S*, as:
(1)
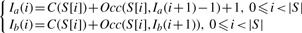

where *I_a_*(*i*) and *I_b_*(*i*) represent the starting and end indices of the *SA* interval for the suffix of *S* starting at position *i*, and *I_a_*(|*S*|) and *I_b_*(|*S*|) are initialized as 0 and |*T*|, respectively. The calculation stops if it encounters *I_a_*(*i*+ 1)> *I_b_*(*i*+ 1). The condition *I_a_*(*i*) ≤*I_b_*(*i*) holds if and only if the suffix of *S* starting at position *i* is a substring of *T*. The total number of the occurrences is calculated as *I_a_*(0) − *I_b_*(0) + 1 if *I_a_*(0)≤*I_b_*(0), and 0, otherwise. After getting the *SA* interval, the location of each occurrence can be determined by directly looking up *SA* with a constant time complexity. Hence, the time complexity for finding *n* occurrences of *S* is*O*(|*S*|+*n*).

#### 2.1.3 Reducing memory overhead

From Equation (1), the substring search using the FM-index does not require *B_T_*, meaning that the overall memory footprint is the memory sum of the FM-index and *SA*. This memory overhead (~60 GB for the human genome) can be reduced by an order of magnitude by taking advantage of some features of the BWT at the cost of a slightly higher substring search time complexity as follows.

For the FM-index, *Occ*(•) dominates the overall memory overhead. An approach to trade-off speed and memory space is to use a reduced FM-index (detailed in the Supplementary Material) with a time complexity of *O*(*u*·|*S*|) for substring search, which is able to reduce the memory size to |Σ|⌈log_2_ |Σ|⌉ + |*T*|(|Σ|⌈log_2_ |*T*|⌉ */u* + ⌈log_2_ |Σ|⌉) bits (*u* = 128 by default and thus ~1.1 GB for the human genome). For *SA*, by employing the ‘*last-to-first column mapping*’ property of BWT, we can reduce the memory size to |*T*|⌈log_2_ |*T*|⌉ */v* bits (*v* = 8 by default and thus ~1.5 GB for the human genome) through the use of a reduced suffix array (detailed in the Supplementary Material) with an approximate time complexity of *O*(*n*·*v*)for locating *n* occurrences of *S*.

Now, we have arrived at a significantly smaller memory footprint of (|Σ| + |*T*|)⌈log_2_ |Σ|⌉ + |*T*|(|Σ| */u* +1*/v*) ⌈log_2_ |*T*|⌉ bits (e.g. ~2.6 GB for the human genome). Furthermore, the increased time complexity*O*(*u*·|*S*| + *v*·*n*) for finding *n* occurrences of *S* is still acceptable.

### 2.2 Estimation of the minimal seed size

We are only interested in the MEM seeds whose lengths are not less than a minimal seed size *Q*. Decreasing *Q* generally increases the sensitivity by finding more hits in homologous regions, at the cost of producing more noisy hits. Increasing *Q* generally decreases the number of hits at the cost of decreased sensitivity. Many seed-based aligners, therefore, require users to carefully tune *Q*. However, this tuning work is tedious. To address this issue, we propose an automatic estimation of *Q* according to a given read length.

Our estimation of *Q* is based on the *q*-gram lemma (Rasmussen *et al*., 2006) and a simplified error model. The *q*-gram lemma states that two aligned sequences *S*_1_ and *S*_2_ with an edit distance of *e* (the number of errors) share at least *t q*-grams where *t* = max(|*S*_1_|,|*S*_2_|)−*q*+1−*q*·*e*. This means that for overlapping *q*-grams, one error may cause up to *q*·*eq*-grams not to be shared by the two reads, and for non-overlapping *q*-grams, one error can destroy only one *q*-gram (Blom *et al*., 2011). Hence, given the edit distance *e* of *S* aligned to the genome, *Q* is estimated as:
(2)
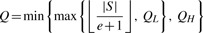

where *Q_L_* and *Q_H_* are the global lower-bound and upper-bound, respectively. The estimation is based on the pigeonhole principle for non-overlapping *q*-grams, meaning that at least one *q*-gram of length *Q* is shared by *S* and its aligned substring mate on the genome. By default, our aligner sets *Q_L_* = 13 and *Q_H_* = 49.

Since the error model for gapped alignments is quite complicated, we employ a simplified error model for ungapped alignments to estimate *e*. Supposing that the number of substitutions *w* in the full-length alignment of *S* is a random variable and each base in *S* has the same error probability *p* (default = 2%), the probability of having *z* substitutions is calculated as:
(3)
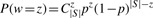

where *w* follows a binomial distribution. By specifying a missing probability *m* (default = 4%), *e* can be estimated as min{*z*|*P*(*w > z*)*< m*}. Our simplified error model results in the following values: *Q* = 16 for 100-bp reads, *Q* = 22 for 200-bp reads and *Q* = 35 for 500-bp reads. In addition, we also provide parameters to allow users to customize *Q*.

### 2.3 Generation of maximal exact matches

To identify MEMs between *S* and *T*, we advance the starting position *p* in *S*, from left to right, to find the longest exact matches (LEMs) using the BWT and the FM-index. According to the above definitions, we know that the identified LEMs are right maximal. We know that the LEMs starting at the beginning of *S* are both left maximal and right maximal. This means that when advancing the starting positions from the beginning to the end of *S*, the identified LEMs are also left maximal if it is not part of any previously identified MEM. In this way, only unidirectional substring search is required. Since we are only concerned about MEMs of sufficient lengths, we discard the MEMs whose lengths are less than *Q*. For large genomes, it is possible to find a lot of occurrences of a MEM starting at a certain position of *S*. In this case, we only keep its first *h* (*h*= 1024 by default) occurrences and discard the others.

However, it is also observed that we sometimes fail to find any MEM seeds for some reads using *Q*. To improve sensitivity, we therefore attempt to rescue them by re-conducting the MEM identification procedure using a new and smaller minimal seed size *Q_N_* = (*Q* + *Q_L_*)*/*2.

### 2.4 Determination and selection of mapping regions

For local alignment with affine gap penalty, the positive score for a match is usually smaller than the penalty charged for a substitution or for a gap. Using such type of scoring schemes, the length of the optimal local alignment of *S* to the genome cannot be *>*2|*S*| as a local alignment requires a positive alignment score. This conclusion forms the foundation of our genome mapping region determination approach for each identified MEM seed. In our aligner, we employ a commonly used scoring scheme [e.g. also used in BLAST (Altschul *et al*., 1990) and BWA-SW] with the score 1 for a match, a penalty of 3 for a substitution, a penalty of 5 for a gap opening and a penalty 2 for a gap extension.

For a read, a MEM indicates a mapping region on the genome, which includes the seed and potentially contains the correct alignment of the full read. We can determine the range of the mapping region by extending the MEM in both directions by a certain number of bases. Since the optimal local alignment length of *S* cannot be *>*2|*S*| in our aligner, it is safe to determine the mapping region range by extending the MEM by 2|*S*| bases in each direction. This extension does work, but will result in lower speed due to the introduced redundancy. Hence, we attempt to compute a smaller mapping region with as little loss of sensitivity as possible.

We define *P*_mem_ to denote the starting position of a MEM in *S*, *T*_mem_ to denote the mapping position of the MEM on the genome and *L*_mem_ to denote the MEM length. Assuming that the MEM is included in the final alignment, our aligner estimates the mapping region range [*T_a_*, *T_b_*] as:
(4)
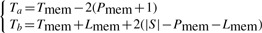

Our aligner computes the optimal local alignment scores in all determined mapping regions of *S* using the SW algorithm, and then builds a sorted list of all mapping regions in the descending order of score. Mapping regions whose scores are less than a minimal score threshold (default = 30) are removed from the sorted list. Subsequently, the sorted list of qualified mapping regions is used in the SE and the PE alignment (e.g. determining the final alignments and approximating the mapping quality scores).

### 2.5 Paired-end mapping

The alignment of a paired read pair generally has two constraints: *alignment strand* and *mapping distance*. For the alignment strand constraint, our aligner requires the two reads to be aligned to the genome from different strands. For the mapping distance constraint, our aligner requires that the mapping distance of the two reads cannot exceed a maximal mapping distance threshold defined by the insert-size information of a library. Assuming that the mean insert-size is 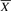 and the standard deviation of the insert-size is *σ*, we calculate the maximal mapping distance threshold as 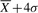. For the PE mapping, our aligner employs two stages: (i) pairing qualified mapping regions in order to find the correct alignments for both ends and (ii) rescuing un-aligned reads through their aligned read mates.

For any aligned read pair, we can first compare their mapping distance on the genome (calculated from the positions of the best alignments of the two reads) to the insert-size constraint. If this comparison is within the mapping distance threshold, the corresponding alignment is output. Otherwise, we could calculate the mapping distance for each mapping position pair from all qualified mapping regions in the sorted list. However, the associated computational overhead cannot be tolerated since we need to obtain the alignment paths for all qualified mapping regions of a read pair. Hence, we introduce a seed-pairing approach to heuristically accelerate the read pairing.

The seed-pairing heuristic works by enumerating each seed pair of *S*_1_ and *S*_2_ in order to find all potential seed pairs. If the seed pair has different alignment strands and locates on the same genome fragment, it will be used to estimate the mapping distance of *S*_1_ and *S*_2_, and otherwise will be discarded. In our aligner, the mapping position *T_s_* of *S* is estimated from one of its MEMs as:
(5)
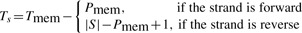

where we assume that *S* is aligned to the genome without gaps. To compensate for the difference between the estimated mapping distance and the correct one, we employ a larger maximal insert-size threshold 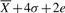 for the seed-pairing heuristic. If the estimated mapping distance does not exceed the maximal insert-size threshold, this seed pair is considered qualified and will be saved for future use. After finding all qualified seed pairs, we enumerate each qualified seed pair to compute the real mapping distance of *S*_1_ and *S*_2_, which is compared with the maximal insert-size threshold 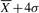. If the insert-size constraint is met, *S*_1_ and *S*_2_ are reported as paired. Otherwise, we will compute the best alignment for *S*_1_ (or *S*_2_) to rescue its mate by employing the insert-size information to determine the potential mapping region of its mate. This rescuing procedure is also applied when only one read of *S*_1_ and *S*_2_ is aligned.

### 2.6 Approximation of mapping quality scores

Since the introduction of mapping quality scores in MAQ (Li *et al*., 2008) to indicate the probability of the correctness of alignments, the concept of mapping quality scores has been frequently used in many NGS read aligners. Generally, a higher mapping quality score indicates a higher confidence in the correctness of an alignment.

As stated in BWA-SW, if an aligner guarantees to find all local alignments of a read, the mapping quality score *M_q_* is determined by these local alignments only. Although our aligner does not find all local alignments of the read, the sorted list of qualified mapping regions still provides sufficient information to approximate *M_q_*. In our aligner, we employ two equations to approximate *M_q_* for the SE and the PE alignment. For the SE alignment, *M_q_* is approximated as 250(*b*_1_ − *b*_2_)/*b*_1_ × *r*, similar to the mapping quality approximation in BWA-SW, where *b*_1_ is the best local alignment score, *b*_2_ is the second best local alignment score, and *r* is calculated by dividing the number of bases of the read in the final alignment by the read length. For the PE alignment, the calculation of *M_q_* depends on two conditions. If the two reads are correctly paired through the seed-pairing heuristic, the mapping quality score for each read is equal to its SE *M_q_*. Otherwise, if one read is rescued by its mate, the mapping quality score of the read is approximated as *r* × *M*_mq_, where *M*_mq_ is the SE *M_q_* of its mate.

### 2.7 Parallel design

In CUSHAW2, the most time-consuming part of SE alignment is the selection of the best mapping region using the SW algorithm. To accelerate its execution, we have adopted the (SSE2) Streaming SIMD Extensions 2-based parallel implementation of the SW algorithm in SWIPE (Rognes, 2011).

In addition, as multi-core CPUs have become commonplace, our aligner employs a multi-threaded design using *Pthreads* to parallelize the alignment process. We use a dynamic scheduling policy to assign reads to threads, which allows one thread to immediately start a new alignment without waiting for the completion of the other threads. For SE alignment, a thread aligns a single read at a time and then reads a new read from the input file immediately after finishing the current alignment. For PE alignment, we follow the same scheduling policy with the difference that one read pair is assigned at a time. Locks are appropriately used to ensure mutually exclusive accesses to both the input and output files.

## 3 RESULTS

The performance of CUSHAW2 is compared with three other long read aligners: BWA-SW (v0.6.1), Bowtie2 (v2.0.0-beta5) and GASSST (v1.28). BWA-SW employs the default settings and Bowtie2 also employs the default settings, except for the insert size related parameters for the PE alignment. GASSST uses a minimal percentage identity of 90% and default settings for other parameters. CUSHAW2 requires the final alignment to have a percentage identity of ≥90% (default setting) and to include ≥80% (default setting) bases of the read.

All the tests are conducted on a workstation with two six-core Intel Xeon X5650 2.67GHz CPUs and 96 GB RAM, running the Linux operating system. The runtime of each aligner is measured in wall clock time for all tests, where the one-time construction time of the BWT and the FM-index is not counted in for CUSHAW2, BWA-SW and Bowtie2. We use the recall and precision measures to assess all aligners using simulated datasets, where recall (precision) is calculated by dividing the number of reads that are correctly aligned by the total number of reads (the number of aligned read). If not explicitly specified, a read is deemed to be correctly aligned if the mapping position has a distance of ≤5 to the true position. For real datasets, we use the sensitivity measure, which is calculated by dividing the number of aligned reads by the total number of reads.

GASSST does not evaluate every seed to determine the best alignment for a single read. Instead, it continues reporting identified alignments until reaching the maximal limit of the number of alignments. Thus, we consider the best of all reported alignments as the final alignment of the read and discard the others. In addition, GASSST does not provide the support for PE alignment and thus is only evaluated for SE alignment. BWA-SW might report more than one alignment for a single read (in rare cases for not very long reads), where one alignment corresponds to one fragment of the read. Since these fragment alignments are difficult to be distinguished and ranked, we take all of them into consideration.

### 3.1 Evaluation on simulated datasets

We have first evaluated all aligners using nine simulated 100-bp, 200-bp and 500-bp datasets with different uniform base error rates (i.e. 1%, 2% and 4%). These datasets are simulated from the human genome using the *wgsim* utility in SAMtools v0.1.17 (Li *et al*., 2009) with 10% errors being indel errors. Each dataset is comprised of 2 million PE reads, and the insert-sizes are drawn from normal distributions *N* (500, 50), *N* (1000, 50) and *N* (2000, 50) for the 100-bp, 200-bp and 500-bp datasets, respectively.

Table 1 shows the alignment results of all aligners for the 200-bp datasets, whereas the alignment results for the other datasets can be obtained from the Supplementary Material. CUSHAW2 yields the highest recall and precision for both the SE and the PE alignment for all datasets (with an exception that for the 100-bp dataset with 1% error rate, Bowtie2 has a slightly better precision than CUSHAW2 by ~0.04% for the PE alignment). Furthermore, CUSHAW2 is on an average superior to all other evaluated aligners in terms of both recall and precision for the SE (PE) alignment, where the average recall is ~89.92% (90.75%) and the average precision is ~97.46% (98.28%). For the SE alignment, on average, BWA-SW is the second best with an average recall (precision) of ~88.54% (96.81%), whereas GASSST is the worst with an average recall (precision) of only ~79.46% (95.51%). For the PE alignment, on average, BWA-SW has a higher recall than Bowtie2, whereas the latter gives a higher precision. For BWA-SW, the average recall (precision) is ~90.14% (97.65%) and for Bowtie2, ~89.71% (97.92%). Moreover, an increased recall and precision can be observed for CUSHAW2, BWA-SW and Bowtie2 after performing PE mapping on each dataset. In general, for each aligner, both recall and precision improve for increasing read length for a fixed error rate, and degrade for increasing error rates for a fixed read length.

**Table 1.** Alignment results on the simulated 200-bp datasets

Aligner	1%		2%		4%	
	Recall	Prec.	Recall	Prec.	Recall	Prec.
	SE					
CUSHAW2	**90.39**	**97.84**	**90.28**	**97.77**	**90.02**	**97.57**
BWA-SW	90.30	97.74	90.03	97.50	88.92	96.43
Bowtie2	89.99	97.45	89.44	96.97	87.41	96.03
GASSST	80.41	96.04	79.58	96.01	77.73	95.98

	PE					
CUSHAW2	**90.94**	**98.44**	**90.85**	**98.39**	**90.76**	**98.26**
BWA-SW	90.51	97.97	90.42	97.90	90.19	97.51
Bowtie2	90.82	98.32	90.48	98.03	89.16	97.58

To evaluate alignments with high mapping quality scores, we have taken into account the alignments whose mapping quality scores are ≥30 (Q30). Moreover, an aligned read is deemed to be correctly aligned only if the mapping position is identical to the true position of the read. Table 2 shows the alignment results using Q30 for the 200-bp datasets, whereas the alignment results using Q30 for the other datasets can be obtained from the Supplementary Material. For both the SE and the PE alignment, on average, CUSHAW2 yields the highest recall whereas Bowtie2 gives the highest precision. For all datasets, the average recall (precision) for SE alignment is ~85.80% (99.94%) for CUSHAW2, 81.70% (99.94%) for BWA-SW, 76.47% (99.98%) for Bowtie2 and 75.44% (99.51%) for GASSST. On an average for all datasets, the recall (precision) for PE alignment is ~86.01% (99.94%) for CUSHAW2, 84.15% (99.94%) for BWA-SW and 81.84% (99.98%) for Bowtie2. Compared with the SE alignment, the recall of each aligner gets better for each dataset. As for the precision, both CUSHAW2 and BWA-SW can hold their precision for each dataset, whereas Bowtie2 has a minimal decrease for some datasets.

**Table 2.** Alignment results using Q30 on the simulated 200-bp datasets

Aligner	1%		2%		4%	
	Recall	Prec.	Recall	Prec.	Recall	Prec.
	SE					
CUSHAW2	**86.32**	99.95	**86.07**	99.94	**85.95**	99.93
BWA-SW	85.80	99.94	85.04	99.94	82.33	99.93
Bowtie2	80.76	**99.98**	76.96	**99.98**	71.59	**99.98**
GASSST	76.15	99.54	75.47	99.57	73.92	99.49
	PE					
CUSHAW2	86.34	99.95	**86.12**	99.94	**86.17**	99.93
BWA-SW	**86.55**	99.95	86.09	99.94	84.25	99.93
Bowtie2	83.81	**99.98**	82.71	**99.97**	82.71	**99.97**

Finally, we have evaluated the impact of the amount of indel errors on each aligner. In this evaluation, we have re-simulated four 200-bp datasets from the human genome containing 2 million PE reads each. All the four datasets have the same uniform base error rate of 2%, but have different percentages of indel errors (i.e. 20%, 40%, 60% and 80%). Table 3 shows the alignment results. For the SE alignment, CUSHAW2 has the highest recall and precision for all datasets. BWA-SW is second and GASSST is worst. For the PE alignment, CUSHAW2 still holds the highest rank in terms of both measures. For each aligner, both the recall and the precision nearly keep constant with negligible fluctuations. This suggests that every aligner can tolerate a high percentage of indel errors.

**Table 3.** Alignment results using different percentages of indel errors

Aligner	Measure	20%	40%	60%	80%
	SE				
CUSHAW2	Recall	**90.29**	**90.26**	**90.27**	**90.29**
	Prec.	**97.77**	**97.76**	**97.77**	**97.76**
BWA-SW	Recall	90.05	90.05	90.03	90.05
	Prec.	97.49	97.52	97.50	97.49
Bowtie2	Recall	89.45	89.43	89.45	89.46
	Prec.	96.96	96.97	96.99	96.97
GASSST	Recall	79.61	79.55	79.57	79.59
	Prec.	96.01	96.01	96.04	96.04

	PE				
CUSHAW2	Recall	**90.87**	**90.83**	**90.84**	**90.86**
	Prec.	**98.38**	**98.38**	**98.38**	**98.37**
BWA-SW	Recall	90.43	90.41	90.41	90.44
	Prec.	97.89	97.90	97.90	97.90
Bowtie2	Recall	90.5	90.49	90.49	90.51
	Prec.	98.03	98.05	98.04	98.04

### 3.2 Evaluation on real datasets

We have assessed all aligners using four datasets produced by 454 and Illumina sequencers, respectively. All the datasets are publicly available from NCBI SRA and named after their accession numbers (see Table 4). We have used two runs (SRR000026 and SRR000027) of the SRX000001 experiment, two runs (SRR006428 and SRR006433) of SRX001829, two runs (ERR024139 and ERR024140) of ERX009608 and the single run (SRR189815) of SRX028059. For all 454 datasets, we have removed all reads shorter than 100-bp and only conducted the SE alignment. For all Illumina datasets, we have performed both the SE and the PE alignment.

**Table 4.** Real dataset information

Type	Name	No of Reads	Max	Mean	Insert
454	SRX000001	1 026 049	849	192 ± 58	–
	SRX001829	2 790 032	4996	560 ± 165	–

Illunima	ERX009608	107 967 800	102	101 ± 1	311
	SRX028059	243 441 880	102	101 ± 1	510

**Fig. 2.**
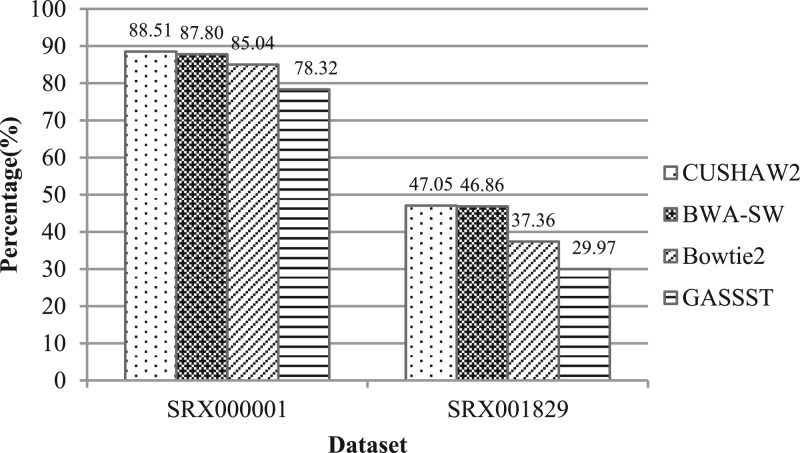
Alignment results using the 454 datasets

**Fig. 3.**
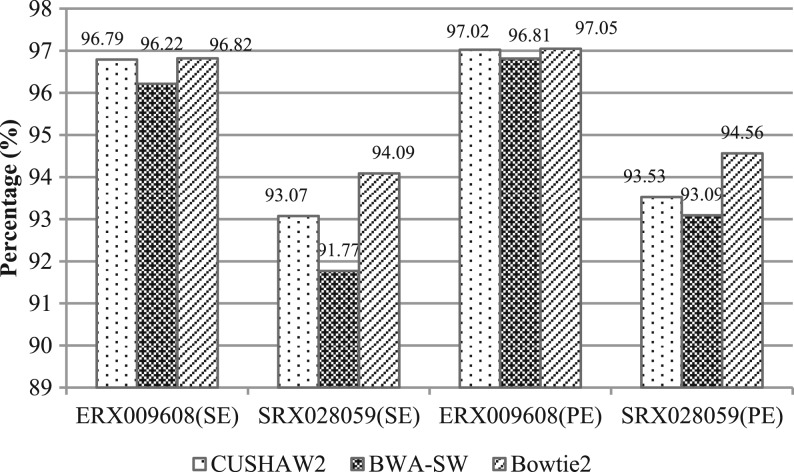
Alignment results using the Illumina datasets

Figures 2 and 3 show the alignment results for the 454 and Illumina datasets, respectively. In this evaluation, the alignment of a read is taken into account only if it has a percentage identity of ≥90% and contains ≥80% bases of the read. This constraint is also in accordance with our intention for long read alignments, i.e. attempting to align a read in the full length to the genome. We have excluded GASSST from the larger Illumina datasets due to its very slow speed.

For each 454 dataset, CUSHAW2 has the highest sensitivity. BWA-SW is second and GASSST is worst. For the Illumina datasets, Bowtie2 aligned the most reads and CUSHAW2 the second most. For the SE (PE) alignment, the average sensitivity is ~94.93% (95.28%) for CUSHAW2, 93.99% (94.95%) for BWA-SW and 95.45% (95.81%) for Bowtie2.

### 3.3 Speed and scalability evaluation

We have assessed the speed of each aligner using 12 threads on our workstation (see Table 5), where we organized all simulated datasets into three groups, namely D100, D200 and D500, as per the read lengths and averaged the runtimes of all datasets in each group.

**Table 5.** Runtime comparison (in seconds) of the tested aligners

Data Group	CUSHAW2	BWA-SW	Bowtie2	GASSST
	SE				
D100	108	116	64	3348
D200	238	302	147	3538
D500	1157	842	2,038	4574
SRX000001	57	87	40	3273
SRX001829	925	758	1796	3941
ERX009608	2499	3761	1909	–
SRX028059	11 551	16 731	8191	–
	PE				
D100	110	123	77	–
D200	241	320	240	–
D500	1157	928	2179	–
ERX009608	2657	4053	2048	–
SRX028059	12 936	17 093	9741	–

For the SE alignment, GASSST is the slowest for each data group and is almost two orders of magnitude slower than the other three aligners for D100, D200 and SRX000001. For the datasets with smaller mean read lengths of ≤200-bp, Bowtie2 is the fastest and CUSHAW2 outruns BWA-SW. However, for the datasets with greater mean read lengths of around 500-bp, Bowtie2 becomes slower than both CUSHAW2 and BWA-SW and BWA-SW outruns CUSHAW2. This suggests that Bowtie2 might have been optimized for reads of lengths around 200-bp, but does not scale well towards longer reads. The PE alignment comparison between CUSHAW2, BWA-SW and Bowtie2 shows a similar trend.

From the runtimes of all simulated data groups, it is observed that the runtime for CUSHAW2, BWA-SW and Bowtie2 significantly increases as the read lengths grow from 100 to 500. This can be explained by the quadratic time complexity of the DP-based alignment computation and the increasing number of seeds for longer reads. However, the runtime of GASSST does not increase significantly for increasing read length due to its use of both the multi-filtration mechanism and the early stop of seed search that stalls when the number of seed occurrences reaches a specified limit (Rizk and Lavenier, 2010). The multi-filtration mechanism is likely to eliminate many noisy seeds efficiently. The early stop of seed search makes the overall number of seeds relatively stable for different read lengths. However, the negative side effect of the two approaches is that it causes the loss of relevant seeds, thus missing some correct alignments as we can observe from evaluation with simulated reads.

**Fig. 4.**
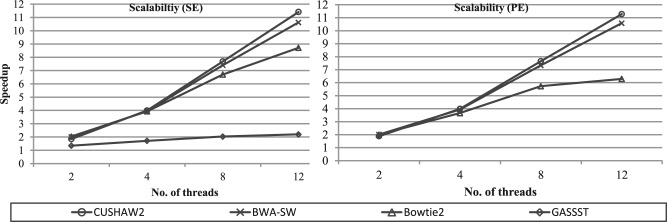
Scalability comparison between all aligners for the SE and PE alignment

Finally, we have assessed the parallel scalability of all aligners with respect to the number of threads. In this evaluation, we have used the three simulated 100-bp, 200-bp and 500-bp datasets with 1% error rate to run each aligner. Figure 4 illustrates the average speedups of each aligner using different number of threads for the SE and PE alignment. For both the SE and PE alignment, CUSHAW2 shows the best scalability and BWA-SW the second best. The scalability of GASSST is the worst for the SE alignment, where it only gets slight speed improvement after doubling the number of threads. Using 12 threads, for the SE (PE) alignment, the average speedup is about 11.4 (11.3) for CUSHAW2, 10.6 (10.6) for BWA-SW and 8.7(6.3) for Bowtie2, whereas GASSST only has an average speedup of about 2.2 for the SE alignment.

## 4 CONCLUSIONS

In this article, we have presented CUSHAW2, a parallel and accurate algorithm and tool for aligning long reads to large genomes, such as the human genome. In this aligner, MEMs are used as seeds to find gapped alignments and final alignments are reported in SAM format (Li *et al*., 2009) to facilitate the downstream analysis. To accelerate the alignment selection, our aligner employs fine-grained parallelism from Single instruction, Multiple data (SIMD) vector execution units with the use of SSE2 assembler instructions. In addition, multithreading is supported in order to benefit from the coarse-grained parallelism on multiple CPU cores.

We have assessed the performance of CUSHAW2 and the three other long read aligners: BWA-SW, Bowtie2 and GASSST using simulated as well as real datasets. For the simulated reads, we have computed the recall and precision measures since we know the true position of each read on the genome. For the real datasets, we have employed the sensitivity measure. Using the above measures, CUSHAW2 is shown to be among the highest-ranked aligners in terms of alignment quality for both the SE and the PE alignment for a variety of error rates and varying amount of indel errors. Our aligner achieves good parallel scalability with respect to the number of threads, while demonstrating highly competitive overall execution speed. Furthermore, through the use of memory efficient data structures, CUSHAW2 only requires a memory footprint of ~4 GB (using 12 threads) for performing alignments to the human genome. This approach makes it possible to accurately align hundreds of millions of long reads to a mammalian-sized genome in only a few hours on a standard multi-core workstation with only a modest amount of RAM installed. Since throughput and read-length of NGS machines continues to grow, these results are of high importance to the bioinformatics community.

*Funding*: We acknowledge funding by the Center for Computational Science, Mainz.

*Conflict of Interest*: None declared.
